# Paediatric Multidrug-Resistant Tuberculosis with HIV Coinfection: A Case Report

**DOI:** 10.1155/2013/756152

**Published:** 2013-01-23

**Authors:** Huldah I. Nwokeukwu, Paulinus N. Okafor, Onuka Okorie, Ihuoma K. Ukpabi

**Affiliations:** ^1^Department of Community Medicine, Federal Medical Centre, PMB 7001, Umuahia, Abia State, Nigeria; ^2^Department of Public Health, Ministry of Health, Abia State, Nigeria; ^3^Department of Paediatrics, Federal Medical Centre, PMB 7001, Umuahia, Abia State, Nigeria

## Abstract

*Background.* Tuberculosis is a major public health problem, and its control has been facing a lot of challenges with emergence of HIV. The occurrence of multidrug-resistant strain has also propounded the problem especially in children where diagnosis is difficult to make. Multidrug-resistant tuberculosis (MDR-TB) is *in vitro* resistant to isoniazid (H) and rifampicin (R). Paediatric multi-drug resistant tuberculosis with HIV coinfection is rare, and there is no documented report from Nigeria. *Objective.* To report a case of paediatric MDR-TB in Nigeria about it. *Methods.* The case note of the patient was retrieved, and relevant data were extracted and summarized. *Results.* A 9-year-old female HIV-positive pupil with a year history of recurrent cough, 3 months history of recurrent fever, and generalized weight loss was diagnosed and treated for tuberculosis but failed after retreatment. She was later diagnosed with MDR-TB and is presently on DOT-Plus regimen. *Conclusion.* Paediatric MDR-TB with HIV co-infection is rare. Early diagnosis and treatment is important to prevent spread of the disease. The use of Isoniazid preventive therapy is recommended for children who come in contact with patients with active tuberculosis and also for HIV patients without active tuberculosis.

## 1. Introduction

Tuberculosis, commonly known as TB, is a contagious and an often severe airborne disease caused by a bacterial infection. TB typically affects the lungs, but it also may affect any other organ of the body. It is usually treated with a regimen of drugs taken from 6 months to 2 years, depending on the type of infection. Multidrug-resistant TB (MDR-TB) is a form of drug-resistant TB in which TB bacteria can no longer be killed by at least the two best antibiotics, isoniazid (INH) and rifampin (RIF), commonly used to cure TB. As a result, this form of the disease is more difficult to treat than ordinary TB and requires up to 2 years of multidrug treatment. Extensively drug-resistant TB (XDR-TB) is a less common form of multidrug-resistant TB in which TB bacteria have changed enough to circumvent the two best antibiotics, INH and RIF, as well as most of the alternative drugs used against MDR-TB. These second-line drugs include any fluoroquinolone, and at least one of the other three injectable anti-TB drugs: amikacin, kanamycin, or capreomycin. As a result, XDR-TB needs up to 2 years of extensive drug treatment and is the most challenging to treat [1]. The emergence and spread of multidrug-resistant tuberculosis (MDR-TB) and extensively drug-resistant tuberculosis (XDR-TB) are major medical and public problems, threatening the global health [2].

Paediatric multidrug-resistant tuberculosis with HIV coinfection is rare in our environment. Although there is no reported case in Nigeria, the presence of acquired resistant strain in paediatric population has been reported in South Africa [3]. MDR-TB can also be defined as strains of* Mycobacterium tuberculosis* that are resistant to Isoniazid and Rifampicin, with or without resistance to other antituberculosis drugs [4, 5].

WHO estimated that out of the 9.4 million cases of tuberculosis worldwide in 2008, 440,000 (3.6%) had MDR-TB. Childhood tuberculosis is estimated at 10%–15% of the total burden, but little is known about the burden of MDR-TB in children [6]. Paediatric MDR-TB accounts for an estimated 15% of all global cases of MDR-TB [7]. The highest TB incidences and HIV prevalence are recorded in Sub-Saharan Africa, and, as a result, children in this region bear the greatest burden of TB/HIV coinfection [3]. Nigeria ranks 10th among the 22 high TB burden countries which collectively bear 80% of the global burden of TB and also takes 3rd position in the global population of persons living with HIV/AIDS. However, HIV infection is the major threat to TB control programme in Africa and calls for a robust and efficient TB/HIV collaboration. 

Pathophysiological and clinical presentations of TB in children differ from what obtains in adults. TB infection in an HIV-infected child has a higher risk not only of progression to disease but also to extra pulmonary dissemination and death [3]. Some factors like host genetics, microbial virulence, and underlying health conditions (e.g., malnutrition) impair immune competence. Anatomical differences in children also modify the presentation of TB compared with adults. Children in close contact with MDR-TB cases are likely to become infected with the same resistant strain [6]. The commonest type of TB in children is smear-negative pulmonary TB.

HIV is associated with decreased chemotaxis, defective granuloma formation, maintenance, and impaired antigen processing and presentation, as well as generalized loss of CD4+ cell and selective clonal depletion of M-TB specific CD4+ lymphocytes. HIV-infected children demonstrate greater morbidity and mortality from TB and also have increased risk of rapid disease progression, unsatisfactory treatment responses and TB recurrence.

MDR-TB is a laboratory diagnosis, and treatment should be empirical according to the drug susceptibility result [6]. Limited availability of centers for culture and drug susceptibility test worsens the problem. Diagnosis of MDR-TB for this patient was made with Gene Xpert (which is very sensitive and specific) [8] and culture. The treatment of MDR-TB is cumbersome, even where second-line drugs are available [9]. Pill burden is also a big challenge, and side effects of the second line drugs are undesirable. 

Treatment of MDR-TB can take 18 to 24 months, and the outcome is poor [9]. The second-line drugs are far more expensive than the first-line drugs. Coinfection with HIV poses particular challenges and requires early initiation of antiretroviral drugs [6]. The patients are hospitalized during the intensive phase and discharged after sputum conversion to a health center for the continuation phase treatment and monitoring.

DOTS-PLUS recommended by WHO is used in the comprehensive management of MDR-TB. The goal of DOTS-PLUS is to prevent further development and spread of MDR-TB. Treatment regimen: 6(9) Km Ofx Eto Cs ZE + 18Ofx Eto Cs E (KmKanamycin; Ofxofloxacin; Etoethionamide, Cscycloserine, Zpyrazinamide; Eethamabutol) [9]. MDR-TB treatment in children is guided by the same principles using 2nd-line drugs as in adults with careful monitoring of adverse effects [4]. 

The objective of this study is to report a very rare case of pediatric MDR-TB.

## 2. Case Report

A 9-year-old female primary 3 pupil presented at Federal Medical Centre, Umuahia, Abia State, South Eastern Nigeria, with a year history of recurrent cough (initially productive of bloody sputum but later became dry), 3 months history of recurrent fever, and generalized weight loss. There was no history of contact with a patient with pulmonary tuberculosis. The mother tested positive to HIV, while the blind father was HIV negative. On examination she was chronically ill looking and pale with generalized muscle wasting and hypopigmented spots.

Investigations carried out included retroviral test, CD4, sputum acid-fast bacilli and chest X-Ray. The retroviral test was positive, and the CD4 count was 543 cells/uL. The chest X-ray was suggestive of pulmonary TB. The sputum acid-fast bacilli (AFB) result at presentation was 0, +. The diagnosis of retroviral disease was made first and pulmonary tuberculosis a month later. The patient was started on antituberculosis drug with category one regimen which includes rifampicin, isoniazid and pyrazinamide for 2 months and rifampicin and isoniazid for 4 months (2RHZ/4RH). After five months, the patient's sputum AFB was still positive and was declared a failure case. The category two regimen was then started with two months of streptomycin, rifampicin, isoniazid and pyrazinamide, one month of rifampicin, isoniazid, and pyrazinamide and 5 months of rifampicin and isoniazid (2SRHZ/RHZ/5RH) concurrently with antiretroviral drugs (zidovudine, lamivudine and efavirenz). However, at five months, the patient was still sputum AFB positive with results as follows: 6/100 and + and CD4 count of 268 cells/uL, and the patient was declared as failure case after retreatment. The patient was suspected as a case of multidrug-resistant TB and the sputum was collected and sent to a centre in Ebonyi State, South Eastern Nigeria, where there is instrument for investigation (Gene Xpert). The Gene Xpert was done and the result of multidrug resistant tuberculosis was positive. Since multi drug resistant cases are not managed in this particular centre the patient was referred to University College Hospital (UCH), Ibadan, South Western Nigeria, where facilities for management of MDR-TB cases are available and culture and DST were done to confirm the diagnosis. The patient is presently receiving treatment in UCH and responding very well. At the time this patient was referred to this centre, it was confirmed as the first paediatric multidrug-resistant case seen and needed to get the regimen for the management. 

## 3. Discussion

Multidrug-resistant tuberculosis is rare in children [10], and this happens to be the first reported case in Nigeria. It could be that there has not been enough awareness creation about multidrug-resistant tuberculosis especially in children; it could also be that the case search is not yet intensified. In children it is difficult to make a diagnosis of tuberculosis especially MDR. Many children visit the clinic many times before the diagnosis of tuberculosis, and this could be because there is no high suspicion of the disease in the children. It is expected that if the algorithm for TB screening in children where you screen for TB with any of the following symptoms: poor weight gain, fever, current cough, and contact with TB case is used, more of the cases will be detected early. Sub-Saharan Africa bears the brunt of the HIV-fuelled TB epidemic. The rapidly increasing HIV epidemic could also increase the number of HIV-related TB cases [8] and also increase in the MDR-TB.

Multidrug-resistant tuberculosis could be due to primary infection, acquired infection, and amplified resistance. The acquired MDR-TB can be caused by incorrect prescription, irregular supply of drugs, noncompliance of treatment, lack of supervision, loss to followup, and coinfection with HIV while, the primary infection is with a primary MDR strain [9]. This particular patient is most likely to be due to acquired infection since there was no history of contact with a case of MDR-TB or any patient with pulmonary TB. HIV is not only a major risk factor for tuberculosis, but children with HIV are at markedly increased risk of TB [11]. Since the patient is co-infected with HIV, there is the possibility that with this reduced immunity, there will be poor response to the treatment. In a resource-limited setting like Nigeria, where treatment of TB depends on donor agencies for provision of drugs and equipment for investigation, this constitutes a big challenge in the management of tuberculosis.

The diagnosis of HIV was made, and within one month, TB was also discovered in the patient. This showed that there was little or no gap for the use of isoniazid preventive therapy on the child to prevent tuberculosis. There are problems of the use of three I's (intensified case search, infection control, and isoniazid preventive therapy) in this particular patient. If there was intensive case search, there would have been early detection and use of isoniazid preventive therapy. However, with the late detection, if there was infection control, the patient would not have been infected with tuberculosis. Although it seems that the patient was already infected with both HIV and TB before reporting to the hospital, prevention of coinfection in a child of mother with HIV should not have gone unnoticed if the mother had done voluntary counseling and testing. It was noted that diagnosis of TB in this patient was made with sputum AFB which is not usually the case in children especially in coinfection with HIV.

The management of this patient also revealed some of the things needed in such a tertiary intuition where HIV and TB patients are treated. To be able to make the diagnosis of MDR-TB, the sputum was taken to a health facility in another state of Nigeria causing so much delay. For conformation and management, the case was also taken to another state of the country, thereby leading to patient coming in contact with so many people that may lead to the spread of resistant strain, although spread of TB from children is limited.

The CD4 count was 543 cells/uL. and AFB result was 0, + at diagnosis, while by the time the patient was transferred after categories one and two and also on retroviral, the result of CD4 count came down to 268 cells/uL and AFB 6/100(scanty), + which means that the patient deteriorated instead of improving. This could be due to the drugs not being effective since the bacilli were resistant to the drugs. 

## 4. Conclusion

Although the multidrug-resistant tuberculosis is very rare in children, active case search should be instituted especially in high HIV setting and prompt treatment initiated to reduce transmission. Tuberculosis should be controlled with the three I's which are intensified case search, infection control, and Isoniazid preventive therapy. It is imperative, therefore, that children of mothers with HIV should be screened and followed up. If there is HIV in these children, IPT should be given with adequate infection control to prevent TB. It is also recommended that ethambutol which is not used in the management of TB in children because of the optic side effect should be introduced especially in coinfection with HIV to reduce resistance since the benefit outweighs the risk. The Gene Xpert should be made available in all the states of the federation and should not be restricted to only cases that have completed category two. All failure cases even after category one treatment should be screened for early detection and prompt treatment. 

## References

[1] http://www.niaid.nih.gov/topics/tuberculosis/understanding/whatistb/pages/tbdefinitions.aspx.

[2] http://www.hain-lifescience.de/en/products/microbiology/mycobacteria/facts-about-resistant-tuberculosis.html.

[3] Ugochukwu EF (2010). HIV/TB co-infection in Nigerian Children. *Nigerian Medical Journal*.

[4] World Health Organization Nigeria Country Programmes. AIDS, TB and Malaria. http://www.afro.who.int/.

[5] World Health Organization (2004). *TB/HIV. A Clinical Manual*.

[6] Schaaf HS, Marais BJ (2011). Management of multidrug-resistant tuberculosis in children: a survival guide for paediatricians. *Paediatric Respiratory Reviews*.

[7] Ettehad D, Schaaf HS, Seddon JA, Cooke GS, Ford N (2012). Treatment outcomes for children with multidrug-resistant tuberculosis: a systematic review and meta-analysis. *The Lancet Infectious Diseases*.

[8] Cepheid Xpert MTB/RIF. Two hours detection of MTB and resistance to rifampicin. http://www.cepheidinternational.com/tests-and-reagents/ce-ivd-test/xpert-mtbrif.

[9] Park K (2011). *Park's Textbook of Preventive and Social Medicine*.

[10] Shingadia D, Novelli V (2003). Diagnosis and treatment of tuberculosis in children. *Lancet Infectious Diseases*.

[11] http://www.who.int/tb/challenges/hiv/en/index.html.

